# Validation of the menstrual sensitivity index in adolescents

**DOI:** 10.1097/PR9.0000000000001315

**Published:** 2025-08-07

**Authors:** Laura C. Seidman, Ariel B. Handy, Laura A. Payne

**Affiliations:** a McLean Hospital, Belmont, MA, USA; b Harvard Medical School, Boston, MA, USA

**Keywords:** Menstrual sensitivity, Menstrual pain, Dysmenorrhea, Scale development, Adolescents

## Abstract

**Introduction:**

The Menstrual Sensitivity Index (MSI), assessing the attunement to and fear of menstrual symptoms, was recently developed and validated in an adult sample.

**Objectives:**

The objective of this study was to validate the MSI in adolescents.

**Methods:**

One hundred forty-one girls aged 13 to 19 participated in a larger parent study on primary dysmenorrhea, and their data were used in the present study. Girls participated at 2 timepoints: baseline (N = 141) and a 1-year follow-up (N = 115). At each timepoint, participants completed the MSI and a variety of self-report measures.

**Results:**

A confirmatory factor analysis supports the previously identified factors of the MSI (somatic anxiety, fear/danger, and medication) in adolescent girls. The MSI demonstrates good item-total correlations, internal consistency, and test–retest reliability. The MSI converged most strongly with pain catastrophizing and diverged most strongly from body pain.

**Conclusion:**

The MSI is a valid and reliable measure of attunement to and fear of menstrual symptoms in adolescents. Unlike research in adult women demonstrating convergence between the MSI and menstrual pain and menstrual symptom severity, the divergence between the MSI and these variables in the current study suggests that, for adolescents, menstrual sensitivity is more strongly related to fear of pain than menstrual pain itself. These findings provide considerations for future research and clinical practice addressing menstrual pain and symptoms in adolescents.

## Abstract 2

Supplemental Digital Content is Available in the Text.

The Menstrual Sensitivity Index assesses the attunement to and fear of menstrual symptoms and is valid and reliable for use in adolescents.

## 1. Introduction

Dysmenorrhea is defined as pain during menstruation that is not attributable to specific pelvic pathology (primary dysmenorrhea) or that is related to an underlying condition such as endometriosis (secondary dysmenorrhea).^11^ Results from a recent meta-analysis indicate that dysmenorrhea is highly prevalent, such that, cross-culturally, 72.5% of school-aged girls and 74.9% of university-aged women report being affected by this condition.^1^ In part due to this high prevalence, the societal effects of dysmenorrhea are profound. For school-aged youth, dysmenorrhea is associated with reductions in classroom attendance, performance, social activities, and extracurriculars such as sports.^1,3^ Among adults with dysmenorrhea, it is estimated that 140 million working hours are lost annually in the United States.^43^ In Japan, annual economic losses are estimated at ¥683 billion (US $8.6 billion), namely related to absences, decreased work volume, and decreased work efficiency.^56^ Dysmenorrhea also increases susceptibility to developing additional chronic pain conditions.^19,20,29^ Despite the high prevalence and large societal and health effects of dysmenorrhea, this condition is often poorly treated,^29^ yielding a strong need for understanding the mechanisms perpetuating this condition in girls and women.^42^ (We have used the terms “girls” and “women” throughout the manuscript but acknowledge that not all individuals who menstruate identify as girls or women. Gender identity was not assessed in the current study and was not included in any inclusion or exclusion criteria.)

Recently, the Menstrual Sensitivity Index (MSI) was developed and subsequently validated for use in adult women. The MSI assesses the construct of menstrual sensitivity (MS), ie, “the fear and anxiety related to physical symptoms occurring specifically as a result of menstruation”^22^ and consists of 3 factors: somatic anxiety, fear/danger, and medication. In the initial validation of this scale, MS was found to moderately correlate with ratings of women's menstrual pain, menstrual symptom severity, and pain catastrophizing, suggesting this measure taps into a distinct construct from the experience of menstrual pain. In addition, scores were generally stable across a 3-month follow-up period, suggesting the MSI is assessing a trait-like construct.^22^ Thus, given dysmenorrhea's high prevalence, MS may be a mechanism underlying adult women's experience of dysmenorrhea and, therefore, a viable treatment target, whether due to centralized factors underlying pain perception that would be responsive to cognitive behavior therapy approaches (including mindfulness, decatastrophizing, and coping skills training)^46^ or to hormonal factors influencing brain perception and function.^40,58^ However, this construct has yet to be explored in adolescents, which is the usual time of dysmenorrhea onset.^2^ Validating the measure in adolescents will allow assessment of MS across a broader range of developmental stages, which may yield insight into if (and how) the construct changes over time.

The MSI may represent an important contribution to patient-reported outcomes for dysmenorrhea. A recent systematic review^10^ found that only one of the 19 measures evaluated met criteria for recommended use (the Dysmenorrhea Symptom Interference scale,^6^ which assesses activity limitation due to dysmenorrhea symptoms), and none assessed the construct of MS. More recently, Li et al.^37^ developed a measure of dysmenorrhea catastrophizing, but MS is a broader construct than catastrophizing in that it incorporates both nonpain menstrual symptoms and cognitive appraisals beyond catastrophizing. Psychometrically sound measures assessing all aspects of dysmenorrhea, including MS, are critically needed.

Given the evidence for the interplay among psychological constructs and physical experiences of pain, MS may be of clinical relevance as a mechanism underlying menstrual pain, and it may be experienced differently in adolescents compared to adults. Therefore, MS may represent a possible treatment target in adolescents to improve their quality of life and prevent the transition from cyclic to chronic pain. Accordingly, the aim of the current study was to assess the MSI model fit, reliability, and validity in a sample of regularly menstruating adolescents. We hypothesized that (1) the same 8-item, 3-factor model shown to fit adults would also fit adolescents, and (2) reliability and validity statistics would be the same in this population as was demonstrated in adults. Specifically, that measures of average menstrual pain, menstrual symptom severity, and pain catastrophizing would converge with the MSI, and measures of anxiety sensitivity, anxiety, depression, nonmenstrual body pain, and number of premenstrual syndrome symptoms would not.

## 2. Methods

### 2.1. Participants

**Figure 1.**
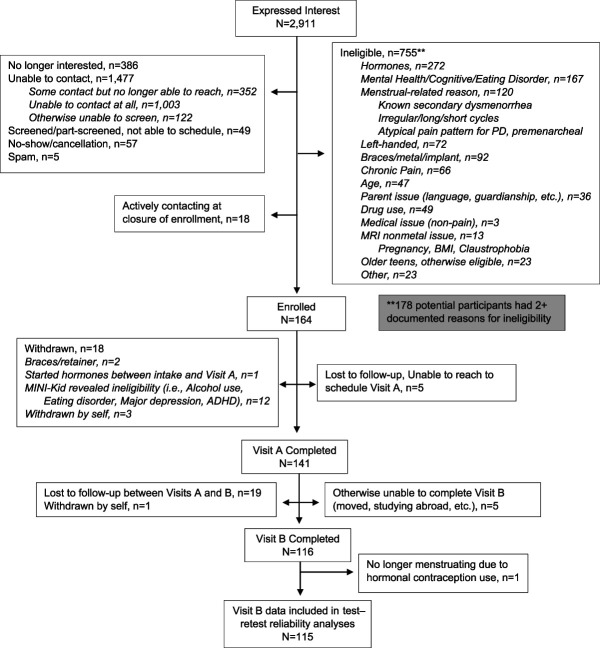
Participant recruitment and enrollment flow diagram. ADHD, attention deficit hyperactivity disorder; PD, primary dysmenorrhea.

Participants included 141 adolescent girls ages 13 to 19 years with varying levels of menstrual pain who were participating in a larger parent study of primary dysmenorrhea in adolescents. See Figure 1 for a flowchart of the recruitment and enrollment process. One hundred sixty-four participants were eligible and enrolled, of which 18 (11.0%) were withdrawn after the intake by themselves (n = 3) or by the principal investigator due to previously unknown eligibility issues (eg, symptoms consistent with an exclusionary medical condition; nondisclosed metal implant; etc.; n = 15). One hundred forty-one participants completed Visit A, of which 116 completed a 1-year follow-up visit (Visit B). Participants were recruited from social media advertisements, a posting on the institution's centralized research recruitment Web site, and word of mouth. Significant efforts were made to recruit a diverse sample regarding race/ethnicity and socioeconomic status representative of the region in which the study took place (eg, social media advertising in a wide catchment area; reimbursement for transportation costs; evening and weekend appointments to allow for parents who are unable to come during business hours, etc.). Recruitment occurred between October 2020 and October 2023; collection of data used in the current analyses occurred between December 2020 and January 2025. Select inclusion criteria included: (1) self-reported menstrual cycle averaging 22–35 days; (2) regular menstrual cycles for at least 6 months; and (3) able to read and understand English. Exclusion criteria included: (1) use of oral contraceptives or any exogenous hormones in the previous 3 months before participation; (2) presence of factors indicative of secondary dysmenorrhea (eg, self-reported presence of persistent pelvic pain throughout the month)^17,21^; (3) diagnosis of chronic pain condition (eg, irritable bowel syndrome [IBS], functional abdominal pain, chronic migraines); (4) current or past diagnosis of any psychotic disorder; (5) currently pregnant; (6) self-reported weekly use of alcohol, cannabis, and/or other illegal substances (due to tasks related to the larger parent study); and (7) developmental delay, diagnosis of autism, or significant cognitive impairment that may preclude understanding of study procedures.

### 2.2. Procedures

The study was conducted at an urban academic medical center in Massachusetts. Potential participants were screened by telephone to assess eligibility. Eligible participants (and a parent for those under 18) were sent a PDF copy of the assent/consent form and instructed to review the form and contact the research team with any questions. Intake visits occurred at any menstrual cycle phase and began with obtaining written informed assent and parent permission from minors (ages 13–17) and a legal guardian; written informed consent was obtained from young adult participants (ages 18–19). Teen participants then used a research team laptop to complete online questionnaires via Research Electronic Data Capture (REDCap).^25,26^ Study visits occurred during the mid-follicular phase (days 8–14 of the menstrual cycle), which was determined based on the most recent first day of menstruation. Study visits included questionnaires completed as described above, an MRI session,^45^ and a subsequent battery of quantitative sensory testing (to be reported elsewhere). Participants were compensated $125 for each study visit. This study was approved by the Mass General Brigham institutional review board (protocol #2019P001729) and was registered on clinicaltrials.gov as NCT04685343.

### 2.3. Measures

#### 2.3.1. Demographics, menstrual history, and body pain

Demographic and menstrual history variables were assessed using an instrument designed for this study. Average menstrual pain without medication was assessed on a 0 (no pain) to 10 (worst pain possible) numeric rating scale (NRS).^5,13,59^ Participants rated 14 separate menstrual symptoms (eg, bloating, headache, constipation, etc.) on 0 (not present) to 10 (extremely severe) NRSs,^7^ which were totaled into a composite variable ranging from 0 to 140. Symptoms of premenstrual syndrome (PMS) were assessed using a multi-checkbox item allowing participants to endorse the symptoms they usually experience before the onset of menstruation but that are no longer present by the end of menstruation. Symptoms were based on a measure developed to aid in the diagnosis of premenstrual dysphoric disorder, but which may be adapted to identify those with PMS^14^; examples include feeling out of control, feeling worthless or guilty, having cravings for specific foods, etc. Scores range from 0 to 21. Body pain over the prior month when not menstruating was rated on the 0 to 10 NRS described above.

#### 2.3.2. Menstrual sensitivity

Although the MSI was administered in the original 10-item version, all analyses in this adolescent sample were conducted using the final 8-item version, which was found to have the best model fit in an adult sample.^22^ The MSI is comprised of 3 factors: somatic anxiety (4 items), fear/danger (2 items), and medication (2 items). No response timeframe is given in the measure instructions; participants are asked to answer how strongly they agree or disagree with each statement. Items are rated on a 1 (strongly agree) to 6 (strongly disagree) scale, reverse-scored and recoded (ie, 1–6 becomes 5–0), and values are totaled to create the final measure score. Scores range from 0 to 40 with higher scores indicating greater MS.

#### 2.3.3. Psychosocial functioning

Two 8-item short forms from the Patient-Reported Outcomes Measurement Information System (PROMIS; Pediatric Profile—49 v.2.0) were administered to assess anxiety and depressive symptoms.^30,48^ Items on both scales are rated on 1 (never) to 5 (almost always) scales; items are summed to yield total scores ranging from 8 to 40. Internal consistencies (as marginal reliability) in the initial validation studies^48^ were 0.83 and 0.85 for anxiety and depression, respectively. Internal consistencies in the present sample were α = 0.91 and α = 0.94, respectively.

#### 2.3.4. Pain catastrophizing

The Pain Catastrophizing Scale for Children (PCS-C)^8^ is a 13-item measure assessing the extent to which respondents have catastrophic thoughts in response to pain experiences. Total scores range from 0 to 52. The measure includes 3 subscales: rumination (range 0–16), magnification (range 0–12), and helplessness (range 0–24). Internal consistencies in the initial validation studies were α = 0.87 and α = 0.90^8^ and in the present sample was α = 0.92.

#### 2.3.5. Pain behavior

The 8-item PROMIS Pediatric Pain Behavior scale (Short Form 8a v.1.0) instructs participants to report the extent to which they engaged in certain behaviors (eg, laying down, protecting the part of the body that hurt, etc.) during the prior week when experiencing pain.^9,31^ The original measure was modified to instruct participants to answer according to how they behaved during their most recent period when they had pain. Items are rated on a 1 (Had No Pain) to 6 (Almost Always) scale; items are summed to yield a total score, which ranges from 8 to 48. Internal consistency in the initial validation study was α = 0.92^9^ and for the present sample was α = 0.91.

#### 2.3.6. Anxiety sensitivity

The Child Anxiety Sensitivity Index (CASI)^53,54^ is an 18-item measure assessing anxiety sensitivity (ie, the fear of anxiety symptoms) in children. Items are rated on a 3-point scale (none, some, a lot) and summed to yield a total score, which ranges from 18 to 54. Internal consistency in the initial validation study was α = 0.87^53^ and for the present sample was α = 0.85.

#### 2.3.7. Multisensory sensitivity

The Sensory Hypersensitivity Scale (SHS)^12^ is a 25-item measure assessing sensitivity to various sensory modalities (eg, smell, light, temperature, allergies, pain, etc.). Items are scored on a 1 (strongly disagree) to 5 (strongly agree) scale and averaged to yield a total score. Internal consistency in the initial validation studies ranged from α = 0.76 to α = 0.86^12^ and for the present sample was α = 0.87.

#### 2.3.8. Somatization

The Children's Somatization Inventory (24-item version; CSI-24)^60^ assesses the extent to which respondents experienced different somatic symptoms (eg, dizziness, low energy, pain in limbs, nausea, etc.) during the prior 2 weeks. Items are rated on a 0 (not at all) to 4 (a whole lot) scale and summed to yield total scores ranging from 0 to 96. Internal consistencies in both the initial validation study^60^ and the current sample were α = 0.87.

### 2.4. Statistical analyses

Sample size was determined by the parent study but is comparable to sample sizes of similar measure validation studies.^36,37^ All analyses were conducted within the R environment,^49^ and only participants with complete data as needed for each analysis were used. Scale scores were calculated via mean extrapolation as long as at least 80% of items were answered. One item of 1 participant's Visit B MSI was missing and was, therefore, imputed. The suitability of the data for factor analysis was assessed using the package EFAtools for exploratory factor analyses.^55^ A confirmatory factor analysis (CFA) was then run using the lavaan package for fitting latent variable analysis.^52^ In line with the original development and validation article, the model was run using the same 3 factor structure identified in a sample of adult women.^22^ Model fit was evaluated using the root mean square error of approximation (RMSEA ≤ 0.06), standardized root mean square residual (SRMR ≤ 0.08), CFI (≥0.95), and the χ^2^/df ratio (≤5). ^28,33^ The internal consistency of the MSI was assessed using Cronbach α within the ltm package for latent trait models.^51^ Within the psych^50^ package, test–retest reliability was assessed using intraclass correlation coefficients (ICC) for 2-way random effects, single-rater/measurement (ie, ICC 2, 1). Interpretations of the ICCs include poor (<0.4), fair (0.4–0.6), good (0.6–0.75), and excellent (>0.75).^18^ Spearman rho was used to assess the mean interitem correlation and convergent validity (cutoff = 0.5).^4^ Validated self-report measures were used for all validity analyses and were chosen to match those used in the adult study as much as possible.^22^

## 3. Results

### 3.1. Demographics and menstrual history characteristics

One hundred forty-one participants completed Visit A; of these, 116 girls also completed the 1-year follow-up visit (Visit B). One participant began menstrual-suppressing hormones between Visits A and B and so did not complete the MSI at Visit B. The average length of time between Visit A and Visit B was 12.8 months (*SD* = 1.9, *range* = 11.1–19.8). Participants ranged in age from 13 to 19 years (*M* = 17.2; *SD* = 1.9), the average age of menarche was 12.0 years (*SD* = 1.2), and the average gynecological age was 5.3 years (*SD* = 2.2). There were no significant differences among any of the demographic variables between girls who had reached and completed Visit B and those who had completed Visit A only. See Table 1 for a greater description of the sample.

**Table 1:** Demographic and menstrual cycle characteristics.

Demographic	Whole sample (N = 141)	Baseline + follow-up (N = 115)
	N (%)/Mean (SD)	N (%)/Mean (SD)
Age	17.2 (1.9)	17.2 (1.9)
Race		
White	77 (54.6)	64 (55.7)
Black/African American	14 (9.9)	11 (9.6)
Asian	31 (22.0)	24 (20.9)
American Indian/Alaska Native	1 (0.7)	1 (0.9)
Multiracial	17 (12.1)	14 (12.2)
Does Not identify	1 (0.7)	1 (0.9)
Ethnicity		
Hispanic/Latino	15 (10.6)	11 (9.6)
Non-Hispanic/Non-Latino	126 (89.4)	104 (90.4)
Gynecological age (y)	5.3 (2.2)	5.3 (2.2)
Cumulative menstrual pain exposure (lifetime number of painful periods)		
None	0 (0.0)	0 (0.0)
1–12 periods	30 (21.3)	25 (21.7)
13–24 periods	36 (25.5)	31 (27.0)
More than 24 periods	75 (53.2)	59 (51.3)
Menstrual pain severity	5.4 (2.2)	5.4 (2.2)
Menstrual symptom severity	32.0 (19.1)	31.6 (17.9)
MSI total score*	11.3 (8.5)	11.2 (8.6)

* At visit A.

### 3.2. Confirmatory factor analysis

Before conducting the CFA, the sampling adequacy and, therefore, suitability of the data for factor analysis was assessed using the Kaiser–Meyer–Olkin (KMO) index (cutoff = 0.5).^27^ The KMO index for the present data was 0.842, indicating the data were suitable for factor analysis. Sphericity was assessed using Bartlett test. This test was significant, providing additional support for the suitability of the dataset, *χ*^2^(28) = 4231.28, *P* < 0.001. Maximum likelihood was used to estimate model parameters within the CFA. Overall, model fit was good (RMSEA = 0.111, SRMR = 0.041, CFI = 0.944, χ^2^/df = 2.727). All items mapped onto the same factors as in the adult sample.^22^ Factor 1 (“somatic anxiety”; items 1–4) assesses the anxiety and worry about physical consequences of menstruation. Factor 2 (“fear/danger”; items 5 and 6) assesses the fear and consequences of physical sensations associated with menstruation. Factor 3 (“medication”; items 7 and 8) assesses anxiety about access to medication to alleviate menstrual symptoms.^22^ The range of factor loadings across all 8 items was greater than 0.65, indicating moderate to strong correlations among each item and the factor.^57^ See Table 2 for factor loadings for each item.

**Table 2:** Factor loadings, internal consistency, and reliability statistics for the menstrual sensitivity index.

Item	Factor 1 loadings	Factor 2 loadings	Factor 3 loadings	Item-total correlations	ICC
I often worry about problems in my body related to my period	0.662			0.70	0.52
I have a difficult time enjoying myself because I cannot get my mind off of my menstrual pain/discomfort	0.854			0.86	0.52
As soon as I feel menstrual pain/discomfort, I begin to worry and feel anxious	0.861			0.85	0.55
When I have my period, I am constantly aware of the feelings I have in my body	0.687			0.76	0.56
I often feel that menstrual pain/discomfort could be a sign of a serious illness		0.730		0.66	0.61
When I feel menstrual pain/discomfort, it frightens me		0.749		0.66	0.59
I take medication when I think I'm about to get my period			0.721	0.60	0.46
When I'm about to get my period, I get anxious if I don't have medication available			0.804	0.69	0.77

ICC, intraclass correlation coefficients; MSI, menstrual sensitivity index.

### 3.3. Consistency and reliability

The internal consistency of the MSI was good (α = 0.87). The average interitem correlation coefficient was 0.48, indicating good consistency across items. Regarding test–retest reliability, individual item ICCs ranged from fair (0.46) to excellent (0.77); the ICC of the total score was excellent (0.76). See Table 2 for item-total correlations and test–retest reliability correlations. See Supplemental File 1, http://links.lww.com/PR9/A332 for the interitem correlation matrix.

### 3.4. Validity

Convergent validity was demonstrated most strongly between the MSI total score and measures of pain catastrophizing and pain behaviors during menstruation. The MSI total score diverged most strongly from measures of anxiety sensitivity, depression, and body pain. Regarding the individual factors of the MSI, Factors 1 and 2 aligned most strongly with pain catastrophizing and diverged most strongly from body pain, whereas Factor 3 converged most strongly with pain behaviors and diverged most strongly from both multisensory sensitivity and body pain. See Table 3 for validity statistics.

**Table 3:** Validity statistics for the menstrual sensitivity index.

Measure	Factor 1	Factor 2	Factor 3	MSI total	M	SD
PCSC rumination	0.64	0.46	0.42	0.65	7.94	3.94
PCSC magnification	0.52	0.44	0.29	0.52	1.37	1.78
PCSC helplessness	0.58	0.44	0.33	0.57	4.46	4.21
PCSC total	0.66	0.47	0.43	0.66	13.85	8.94
CSI	0.51	0.43	0.40	0.51	13.35	9.51
PROMIS pain behavior	0.56	0.37	0.51	0.57	24.26	8.66
PROMIS anxiety	0.50	0.34	0.38	0.50	14.96	5.97
CASI*	0.43	0.39	0.25	0.37	30.05	6.16
PROMIS depression	0.33	0.21	0.25	0.39	13.79	6.23
SHS	0.39	0.32	0.23	0.41	2.61	0.59
Nonmenstrual body pain	0.23	0.16	0.23	0.25	2.06	2.01
PROMIS pain interference	0.49	0.35	0.29	0.47	13.55	5.98
Number of PMS symptoms	0.40	0.35	0.32	0.43	6.86	4.31
Menstrual pain	0.43	0.20	0.46	0.45	5.38	2.22
Menstrual symptom severity	0.42	0.22	0.38	0.52	31.95	19.08

* *n* = 44.

CASI, childhood anxiety sensitivity index; CSI, Children's Somatization Inventory; M, mean; MSI, menstrual sensitivity index; PCSC, Pain Catastrophizing Scale—Child Version; PMS, premenstrual syndrome; PROMIS, Patient-Reported Outcomes Measurement Information System; SD, standard deviation; SHS, sensory hypersensitivity scale.

## 4. Discussion

The MSI is a novel instrument validated for use assessing MS, defined as “the fear and anxiety related to physical symptoms occurring specifically as a result of menstruation.”^22^ The current study extends the application of the MSI to adolescents and reports the initial validation and reliability of its use in this population. We hypothesized that model fit, reliability, and validity statistics would be the same in this population compared to what was previously reported in adults. Indeed, confirmatory factor analysis supports the use of the 8-item MSI in adolescent girls, with similar model fit statistics as reported in the initial development and validation of this scale for adult women. Specifically, the model fit statistics for the adolescent population were RMSEA = 0.111, SRMR = 0.041, CFI = 0.944, χ^2^/df = 2.727, and for the adult population were RMSEA = 0.075, SRMR = 0.023, TLI = 0.972, χ^2^/df = 3.54. The degree of MS appears to be higher in adults compared to the present sample of adolescents (M = 24.5 vs 10.2, respectively). Measures of internal consistency and item-total correlations are good, and test–retest reliability is excellent, which continues to support the use of this scale in adolescents.

Different patterns of convergent validity were found between adolescents in the present study and what was initially reported for adults.^22^ Among both populations, the MSI was found to converge with pain catastrophizing, and diverge from measures of anxiety sensitivity, depression, body pain, and number of PMS symptoms. However, for adolescents, the MSI did not correlate as strongly with ratings of average menstrual pain severity (ρ = 0.45) and menstrual symptom severity (ρ = 0.52) as it did in the adult sample (ρ = 0.53 and 0.66, respectively). In other words, although MS and pain catastrophizing tap into a similar construct for girls and women (ie, fear of experiencing pain), MS may be more strongly associated with menstrual symptoms for adults than for adolescents. Longitudinal research examining the time courses of MS throughout adolescence is needed to better elucidate this potential pattern.^20^

Recently, Knox et al.^34^ conducted the first longitudinal study assessing the experience of dysmenorrhea from adolescence into adulthood. In that study, 74 adolescent girls were followed for an average of 10 years. Roughly a quarter (27%) of participants who met diagnostic criteria for dysmenorrhea during adolescence reported no longer experiencing menstrual pain in adulthood.^34^ A number of factors can influence these trajectories, including effective use of nonsteroidal anti-inflammatory drugs, surgical intervention for endometriosis,^23^ or hormonal contraceptive use.^24^ In light of these changes in the course of dysmenorrhea, it is possible that the experience of MS, a mechanism underlying this condition, may be different in adulthood as well.

Although these longitudinal reductions reported by Knox et al.^34^ are meaningful, they also indicate that most adolescent girls with dysmenorrhea continue on to experience dysmenorrhea as adults. This is problematic as adolescent dysmenorrhea is a well-known risk factor for the development of additional chronic pain conditions,^39^ making adolescence a crucial time for intervention. Indeed, adolescent girls and adult women with dysmenorrhea are roughly twice as likely to experience additional chronic pain conditions as those without dysmenorrhea.^38,39^ Using the MSI in primary care and as adolescents transition to specialty gynecologic care could help identify adolescents who may benefit from further intervention targeting MS. This may, in turn, help reduce the persistence of dysmenorrhea into adulthood and the development of additional chronic pain conditions.

The identification of MS builds on previous work indicating that the ways individuals relate to pain influence their experience of pain itself. Pain catastrophizing, which is the tendency to focus on and inflate the likely threat of pain, is a well-documented predictor of pain intensity and is considered a critical treatment target for addressing chronic pain.^47^ This finding also holds true in the context of dysmenorrhea. A recent large-scale, cross-sectional study of adult women with dysmenorrhea indicates that pain catastrophizing positively predicts menstrual pain severity.^15^ This relationship has also been demonstrated in adolescent girls.^44^ These findings align with additional large bodies of research demonstrating that fear of symptoms can increase and/or maintain disorders (eg, panic disorder, irritable bowel syndrome).^35,41^ Relatedly, a cross-sectional study comparing the predictive value of pain catastrophizing on pain intensity and interference (ie, the extent to which pain reduces one's engagement in life activities) in children, adolescents, and adults found that the effect of pain catastrophizing on pain interference was strongest for adolescents.^16^ This means that, on average, fear of pain hinders one's engagement in life activities to the greatest extent during adolescence.

Interestingly, in this population of teens, MS converged with pain behaviors during their most recent period. Teens with higher levels of MS report increased expressions of pain behaviors (eg, laying down, protecting the part of the body that hurt, etc.) during menstruation. This is noteworthy because it may reflect how increased fear of menstrual symptoms results in behavioral changes that may in turn impact pain experience. Future research is needed to explore this relationship in adults.

There are a few limitations to the current study that must be noted. The items in the MSI were initially created and first validated in a sample of adult women and as such may not represent elements of MS that are more salient to adolescents. The diversity of the sample and inclusion of a wide span of adolescent ages contribute to the generalizability of the current findings. However, because the parent study focused on primary dysmenorrhea, it is possible that the results would differ in teens with secondary dysmenorrhea, abnormal uterine bleeding, irregular cycles, etc., or those who have just begun menstruating. Gender identity was not assessed, and it is possible that results may differ in gender minority subgroups. Furthermore, although the sample size was comparable to those of similar studies,^36,37^ it was smaller than the recommended sample size for CFAs (N = 200).^33^ This may reduce the reliability of the results; thus, further investigation of this instrument in adolescents is needed. As with the adult sample, the individual item test–retest reliability in the current sample was questionable, and the use of individual items is not recommended. Furthermore, 2 of the 3 factors each contained only 2 items and, although one- and two-item scales do exist,^32^ it is generally not recommended due to concerns of instability.

In conclusion, the 8-item MSI developed and validated in adults is a reliable and valid measure of MS in adolescents. Unlike research in adult women demonstrating convergence between the MSI and menstrual pain and menstrual symptom severity,^22^ the weaker associations between the MSI and these variables in the current study suggest that MS may be more strongly related to fear of pain than menstrual pain itself at this younger age. More research is needed to elucidate the extent to which MS changes over time, whether changes in MS relate to changes in other aspects of pain or the menstrual cycle, and methods of targeting this construct within the treatment of dysmenorrhea.

## Disclosures

L.A.P. has received consulting fees from Bayer Healthcare, Mahana Therapeutics, and Oregon Health & Science University and speaking fees from Brightside Health, Pacific Rehabilitation Centers, and The Concord Center. This study was supported by a grant from the National Institute of Child Health and Human Development (R01 HD093680; PI: L.A.P.).

## Supplemental digital content

Supplemental digital content associated with this article can be found online at http://links.lww.com/PR9/A332.
